# Fashion informatics of the Big 4 Fashion Weeks using topic modeling and sentiment analysis

**DOI:** 10.1186/s40691-021-00265-6

**Published:** 2021-09-15

**Authors:** Yeong-Hyeon Choi, Seungjoo Yoon, Bin Xuan, Sang-Yong Tom Lee, Kyu-Hye Lee

**Affiliations:** 1 Teaching Fellow, Department of Clothing and Textiles, Hanyang University, Seoul, Korea; 2 Graduate Student, Department of Business Informatics, Hanyang University, Seoul, Korea; 3 Graduate Student, Department of Business Administration, Hanyang University, Seoul, Korea; 4 Professor, School of Business, Hanyang University, Seoul, Korea; 5 Professor, Human-Tech Convergence Program, Department of Clothing and Textiles, Hanyang University, Seoul, Korea

**Keywords:** Fashion informatics, Fashion weeks, Network analysis, Topic modeling, Sentiment analysis

## Abstract

This study used several informatics techniques to analyze consumer-driven social media data from four cities (Paris, Milan, New York, and London) during the 2019 Fall/Winter (F/W) Fashion Week. Analyzing keywords using a semantic network analysis method revealed the main characteristics of the collections, celebrities, influencers, fashion items, fashion brands, and designers connected with the four fashion weeks. Using topic modeling and a sentiment analysis, this study confirmed that brands that embodied similar themes in terms of topics and had positive sentimental reactions were also most frequently mentioned by the consumers. A semantic network analysis of the tweets showed that social media, influencers, fashion brands, designers, and words related to sustainability and ethics were mentioned in all four cities. In our topic modeling, the classification of the keywords into three topics based on the brand collection’s themes provided the most accurate model. To identify the sentimental evaluation of brands participating in the 2019 F/W Fashion Week, we analyzed the consumers’ sentiments through positive, neutral, and negative reactions. This quantitative analysis of consumer-generated social media data through this study provides insight into useful information enabling fashion brands to improve their marketing strategies.

## Introduction

When launching a new clothing product, fashion houses, brands, and designers must analyze the latest fashion trends by investigating the changes in consumer behavior, market environment, and fashion information. The clothing presented in a fashion house’s collection is a conglomeration of the product, target market, and fashion trends, which also reflects the identity of the designer. Despite developments in computer-based text analysis, social media data from sites such as Twitter, Facebook, Instagram, etc. were generally considered incomplete; however, they are presently valued as new sources of marketing information. Consumers use social media to voluntarily express thoughts regarding their experiences, impression of the products, and observations through posts and likes. As social media encourages users to generate new content, they are valuable platforms for businesses to elicit direct responses from the customers about their products.

In the past, most researchers would ask their focus group participants to tweet their thoughts, which were later analyzed (Kendall, 2014). However, social media comments were considered insufficient and unsuitable for a quantitative analysis. Therefore, to compensate its limitations, researchers started including surveys to gather the users’ opinions about products. However, the survey method is also limited because analyzing the information gathered through surveys is extremely time-consuming and the responses that deviate from the questions have to be excluded (An & Park, 2017). Therefore, the research methods used in informatics, such as social network analysis, sentiment analysis, and topic analysis are increasingly receiving greater scholarly attention, especially for analyzing social media content (Hong & Oh, 2016; Jung & Oh, 2016; Lee et al., 2018).

The fashion week is a major fashion event featuring various design features of the latest fashion products. This event is quite complex, as it not only involves clothing, but also a variety of participants from the fashion industry such as fashion houses, designers, models, buyers, celebrities, journalists, and customers (Entwistle & Rocamora, 2006). Therefore, an analysis of the fashion week requires researchers to consider and analyze all these participants using a variety of methods. Many fashion consumers use smart devices, including mobile phones, to search, share, and even create real-time fashion trends (Jennings, 2019). Consumer-driven data enables the fashion industry to explore new ways of forecasting trends and conducting consumer evaluations (Chaudhuri, 2018); therefore, this study uses such data to investigate the consumers’ perception and evaluation of the Big 4 Fashion Weeks, held in 2019.

In particular, this paper aims to analyze the city-wise fashion trends in terms of their design features and customers’ perceptions and evaluations. Our informatics methodology employs three approaches of text-based big data analytics: semantic network analysis, topic modeling, and sentiment analysis, to explore the trends and patterns revealed through the participants’ reactions expressed through tweets regarding the four largest fashion weeks in the world (Paris, Milan, New York, and London). To achieve these goals, our study includes three steps: (1) identifying the keywords that appeared in the 2019 F/W Fashion Week through a social network analysis, (2) a city-wise examination of the themes and topics associated with the collections through topic modeling, and (3) analyzing the sentimental evaluation of the brands that participated in the Fashion Weeks through a sentiment analysis.

This study provides insights on prominent influencers, successful brands and designers, popular fashion trends, and the design features preferred by consumers. Using new informatics approaches, this study contributes toward a more in-depth understanding of the participants from the fashion weeks. This event is particularly relevant because it provides valuable communication opportunities to the key players in the fashion industry, and our findings provide several theoretical and practical implications for the field of fashion communication and marketing.

## Literature review

### Fashion week and latest fashion trends

Fashion is a style of clothing, footwear, accessories, or makeup that is adopted by a wide audience during a particular time (Hidayati et al., 2014). Changes in fashion are important markers for understanding the society, and the “fashion week” is a significant driver of fashion trends in today’s world. A fashion week represents a major event in the fashion industry, which involves fashion designers and brands displaying their collections to the buyers and the media in runway fashion shows (Apparel Search, n.d.). These events influence the trends in the current and the upcoming seasons. The most prominent fashion weeks are held in the four fashion capitals of the world: New York, London, Milan, and Paris. Generally beginning in New York and ending in Paris, the fashion week also known as “the collections,” showcases the upcoming season’s prêt-à-porter clothing (Entwistle & Rocamora, 2006). These types of events are geo-localized within a city or a territory and are scheduled on a public calendar. Accordingly, to broaden the scope of our analysis, this study’s focus area includes fashion weeks from all four cities showcasing their best ready-to-wear collections.

The New York Fashion Week is based on a much older series of events called the “Press Week,” founded in 1943. New York was the first city to begin organizing seasonal shows; however, the very concept of a “fashion show,” originated in Paris (Crenshaw, 2019), which began holding couture shows since 1945 (Wiig, 2017). The Paris Fashion Week was first organized in 1973 under the auspices of the French Fashion Federation (Leaper, 2016). The Milan Fashion Week was founded by the Italian Chamber of Commerce in 1958 (Davies, 2013), and the London Fashion Week was founded by the British Fashion Council in 1984 (Luu, 2009). The Council of Fashion Designers of America created the modern notion of a centralized “New York Fashion Week” in 1993, although cities like London were already using their city’s name in conjunction with the words “fashion week” since the 1980s (Issahaku, 2019). The inter-seasonal collections are called Resort/Cruise (typically showcased before Spring/Summer) and Pre-Fall (typically showcased before the F/W season; Cora, 2018). Traditionally, designers would usually showcase their autumn and winter collections in February and March, and the Spring/Summer collections in September and October (Cafaro, 2020). However, in the recent years, technological advances have changed this schedule as fashion shows have begun to feature garments that are immediately available for sale in both online and physical stores. The present day “see now, buy now” system features clickable videos on their website, where the latest looks are available online immediately, or even during a live show (Pike, 2016).

Several scholars have discussed the importance of analyzing the Big 4 Fashion Weeks. For instance, Zhao and Min (2019) have claimed that analyzing the fashion week can help fashion companies to revolutionize their designs based on the feedback from the analysis. Kim and Lee (2019a, 2019b) explained that the fashion week is a means of communication between fashion brands and the consumers, warranting an in-depth analysis as a valuable communication channel. Additionally, Skov et. al. (2009) claimed that although the fashion week is essential to the fashion industry, it has also become a cultural icon in its own right. Therefore, it is not just an event to showcase fashion trends, but also an opportunity to identify contemporary cultural and social trends. Simultaneously, the fashion week is also a form of visual merchandising where the fashion shows constitutes a major marketing method used at all the levels of the fashion industry. Analyzing the fashion week helps brands to develop new insights about their customers’ preferences and enables them to effectively prepare for the following seasons.

Furthermore, social media is extremely important for communicating planned events such as fairs, exhibits, and festivals to fans, interest groups, and the general population (Brambilla et al., 2017). Social media has greatly enabled consumers to share real-time responses to the fashion week, making their text data (consumer-driven data) more abundant and relevant to the fashion industry. In fact several brands in the fashion industry have used consumers’ social media data to identify upcoming fashion trends (An & Park, 2020) and to evaluate their marketing performance (Heo & Lee, 2019). Accordingly, fashion communication has evolved from a top-down structure to a more democratic form of communication (Zhao & Min, 2019). The fashion week has a variety of implications, wherein a the trickle-down phenomenon popularizes new fashion trends that designers and celebrities present at the fashion week, and the consumers’ opinions of the fashion week in turn provides valuable marketing feedback to the brands.

Traditionally, most analyses concerning fashion trends have used qualitative case studies that were conducted based on the interpretation of the experts. Moreover, such analyses primarily focused on fashion collections, but did not consider the perspectives of consumer preferences or the performance of the fashion brands. The reliability and validity of this approach depended on the personal abilities of the fashion expert group or the researchers. Therefore, using a consumer-driven data approach makes it possible to produce different results with the same data while incorporating individual perspectives. Conversely, in the field of informatics, computer scientists have devoted considerable effort on studying the different methodologies for analyzing and predicting fashion trends (Baskerville & Myers, 2009; Hidayati et al., 2014; Lee et al., 2017; Singh et al., 2019). However, as most of them lacked the background knowledge in fashion, their focus was restricted to technological methods, limiting their ability to generate new insights from their results. Therefore, to address these limitations, fashion researchers have started using big data analytics to assess fashion brands based on their analysis and the predictions of the consumers’ reactions to various fashion collections (e.g., Silva et al., 2019). While these studies were primarily centered on text mining and semantic network analysis, the present study seeks to derive diverse interpretations using more advanced techniques such as topic modeling and sentiment analysis.

### Fashion informatics and applications

Fashion trend analysis has attracted considerable attention due to the rapid of growth of online data, leading to considerable improvements in analysis techniques (An & Park, 2020). Moreover, the topics that appear in social media reflect the interests of the users and form the basis of public opinion (Lee et al., 2014). Therefore, several fashion studies have tapped into the advantages of the increasing accessibility of text mining to analyze consumer responses and fashion trends (e.g., Lang et al., 2020). For instance, Choi and Lee (2020) used the text mining method to determine Korean consumers’ awareness of the new retro fashion (Newtro fashion), and its semantic components. They found that the words “retro,” “new,” “past,” the “1990s,” “reinterpretation,” “big logo,” and “tint glasses” frequently appeared in consumers’ social media posts, indicating major consumer awareness. Lang et. al. (2020) used text mining to investigate the online fashion renting experiences of consumers and identified that the words “easy,” “recommend,” “process,” “site,” “use,” “special,” “highly,” and “navigate” frequently appeared in positive reviews. In another study, Choi and Lee (2021) focused on vegan fashion trends while analyzing the changes in the consumers’ awareness of original and artificial fur and leather, and confirmed that the keywords related to animal protection appeared with high frequency in their posts.

Network analysis is a useful method for obtaining new insights when analyzing the Big 4 Fashion Weeks. The relationships between the keywords (edges) can provide important information and much more objective insight on fashion trends than the previous research conducted by expert groups that used qualitative methods. Studies that have applied network analysis mechanisms to analyze fashion collections and fashion week used the following approaches (An & Park, 2020; Zhao & Min, 2019). Zhao and Min’s (2019) method for analyzing fashion collections using social media involved collecting the Twitter API before, during, and after a Haute Couture show, which was later subjected to a social network analysis. In fact, our study’s analysis of the fashion week referred to the range and the data collection process used by Zhao and Min’s (2019), but used a different method and object of analysis from their study. Additionally, Zhao and Min’s (2019) search keywords were limited to three phrases: “Paris Fashion Week,” “Haute Couture,” and “Chanel Haute Couture,” wherein their research solely focused on Paris’s Haute Couture show to provide an in-depth analysis. In contrast, An and Park’s (2020) study used text mining and a semantic network analysis to determine the prominent design features from fashion trends through a 10-year data from blog posts that included phrases like “jacket” and “fashion collection.” For a trend analysis, they used a time-series cluster analysis to categorize the fashion trends from the blog posts into four clusters: increasing, decreasing, evergreen, or seasonal. Based on the developments in the existing literature, our study asks the following research questions regarding the Big 4 Fashion Weeks.

Research Question 1. What are the prominent keywords derived from a social network analysis of the 2019 F/W Fashion Weeks?

Our study applies the latent Dirichlet allocation (LDA) algorithm to analyze the fashion collections in the F/W Fashion Weeks. This method focuses on recurring patterns of word occurrence from documents to infer the emergent topics. To date, fashion designers, experts, and editors almost unilaterally determine the themes used by fashion collections. However, with topic modeling, even non-fashion experts can identify alternative and informal themes. Based on these advantages, An and Park (2017) used the LDA algorithm to analyze the users’ references to men’s striped shirts. Similarly, Jang and Kim (2017) used the text mining method and the LDA algorithm to analyze the keywords and the research abstracts appearing in the Journal of Fashion Design from 2001 to 2015 to discover the research topics that gained and lost popularity during these years. Based on the aforementioned studies, we concluded that topic modeling techniques are an effective means of extracting a potential classification combining various topics. While some fashion studies have classified and analyzed the fashion trends appearing on social media (e.g., An & Park, 2020), our study incorporates a qualitative analysis to the topic modeling technique used in prior studies and uses its results to categorize the themes followed by the fashion collections. Based on this approach our study proposes a new methodology for the analysis of fashion collections.

Research Question 2. What are the city-wise themes emerging from the topic modeling of the fashion collections from the 2019 F/W Fashion Weeks?

The increase in the amount of the text produced in online environments, especially in social media, and the developments in natural language processing technology have led to considerable interdisciplinary research on sentiment analysis (Lee et al., 2018). Sentiment analysis is mainly used to analyze public perceptions through online textual data, such as movie and product reviews. Lee et. al. (2017) conducted a sentiment analysis of the Amazon fashion product reviews and used a Support Vector Machine (SVM) classifier to build a positive–negative analysis model for analyzing user opinion. Expanding on the original binary method of classifying emotions (positive vs. negative), they used seven categories of emotions to analyze public opinion related to artificial intelligence: anger, aversion, fear, happiness, neutrality, sadness, and surprise, and found that the sentiment of the text determined by these seven categories was different from that determined through the binary method. Heo and Lee (2019) utilized sentiment analysis to analyze trends in the fashion brand Gucci and found discovered a rising brand appreciation since 2015, when Alexandre Michele was hired as creative director. Therefore, we derive the following research question:

Research Question 3. What is the consumer sentiment toward the brands that participated in the 2019 Fall/Winter Fashion Weeks, analyzed through a sentimental evaluation?

## Method

### Data collection and analysis

We used the open Twitterscraper application programming interface (API) in a Python program to extract Twitter’s user data during the Big 4 Fashion Weeks. The data was crawled using Python 3.7, which was then preprocessed and subjected to a morphological analysis. Table 1 shows the field structure. The search keywords used were “#pfw,” “#mfw,” “#nyfw,” and “#lfw,” which refer to the respective fashion weeks from each city: Paris, Milan, New York, and London. The date ranges used for the data collection was limited to the period in which the 2019 F/W Fashion Weeks were held: New York (February 8–16, 2019), London (February 15–19, 2019), Milan (February 19–25, 2019), and Paris (February 25–March 5, 2019). Our data set had a total of 33,525 records.

**Table 1:** Structure of raw data field on Twitter

No.	Field name	No.	Field name
1	Tweet id	11	No. of likes for a tweet
2	Tweet-URL	12	No. of replies for a tweet
3	Tweet text	13	No. of retweets for a tweet
4	Tweet html	14	Username
5	Links inside the tweet	15	User full name/screen name
6	Hashtags inside the tweet	16	User ID
7	Image URLS inside the tweet	17	The tweet is a reply to
8	Video URL inside the tweet	18	A tweet receiving a reply
9	Tweet timestamp	19	List of users the tweet is a reply to
10	Tweet epoch timestamp	20	Tweet ID of the parent tweet

For data preprocessing, we used the natural language toolkit (NLTK) provided by Python 3.7. When tokenizing the data, NLTK module’s Tweet Tokenizer was utilized to improve accuracy and to prevent the tokens from losing their meaning when all punctuations and special characters were removed. Stop-words, such as surveys and suffixes that appeared frequently but were not relevant to the analysis were eliminated using the NLTK’s Stop-words module. During the semantic network analysis we used the NodeXL 1.0.1 program to conduct a centrality analysis, topic clustering, and visualization.

### Text mining

Text mining is a method of extracting information from unstructured text data, such as posts and user comments. It involves the use of natural language processing, text analysis, and computational linguistics to identify and extract information from the source materials (Kim & Kim, 2014). Text mining analyzes a word’s frequency and its probability of occurrence through natural language processing and morphological analysis to identify the links between texts (Callon et al., 1983; Srivastava & Sahami, 2009). Text mining is a form of machine learning, and involves several analysis techniques, including part-of-speech, degree centrality, and the frequency of occurrence analyzes (Feldman & Sanger, 2007). Text mining techniques are both used by themselves (Choi & Lee, 2021) and to provide precedent data for social network analysis or sentiment analysis (e.g., Choi & Lee, 2020; Lang et al., 2020). Likewise, this study applied text mining to provide input for social network analyzes, topic modeling, and sentiment analyses.

### Semantic network analysis

Semantic network analysis is a computer-based social network analysis method that uses messages as its object of analysis rather than people (Mitchell, 1969). It is a method of deriving the characteristics of a network by detecting the strength and regularity of text-based inputs on frequency and concurrency (Park & Leydesdorff, 2004). Within a network, the positions occupied by nodes can be expressed through their centralities (Freeman, 1978; Hanneman & Riddle, 2005; Scott, 2012). The connection centrality, the most employed indicator of the power of a node, refers to the frequency in which one word is connected to another word (Kim & Kim, 2016). Nodes with a high value of median centrality act as bridges connecting groups of nodes within the network. In a semantic network, a word that has a high proximity center tends to be more frequently connected with other words (Bavelas, 1950; Freeman, 1978). The eigenvector centrality is based on the idea that one node connected to another node is important and is determined in proportion to the sum of the centrality values of the nodes directly connected to a particular node. Even if a node has a low centrality, it could still have a high eigenvector centrality, provided that the other connected nodes also have high centralities (Kwahk, 2014). In addition, it can set a theme by grouping keywords with similar characteristics through clustering algorithms on a network analysis program (Clauset et al., 2004).

The centrality measure can be summarized by standardizing the equation as follows: (1) for each calculation, $C_{x} \left( {N_{i} } \right)$ is the centrality of actor *i*; (2) *g* is the number of actors (*i*) in the network (Wasserman & Faust, 1994); (3) the standardized actor—betweenness centrality, is *g* divided by the maximum value ((*g* − 1)(*g* − 2)/2) of the betweenness centrality (Freeman, 1978; Wasserman & Faust, 1994); (4) in the eigenvector centrality, the actor *i* is the *i*th element of eigenvector unit *e*, and *e* presents the largest eigen value of the adjacent matrix, with *x* as an element. *X* is an adjacent matrix with $X_{ij}$ as an element, and $\lambda$ as an array of eigen values (e.g., Bonacich, 2007; Kwahk, 2014).

Degree centrality of actor *i*:

1 $$C^{\prime}_{D} \left( {N_{i} } \right) = \frac{{C_{D} \left( {N_{i} } \right)}}{g - 1}.$$

Betweenness centrality of actor *i*:

2 $$C^{\prime}_{B} \left( {N_{i} } \right) = \frac{{C_{B} \left( {N_{i} } \right) \times 2}}{{\left( {g - 1} \right)\left( {g - 2} \right)}}.$$

Closeness centrality of actor *i*:

3 $$C^{\prime}_{C} \left( {N_{i} } \right) = \left( {g - 1} \right)\left( {C_{C} \left( {N_{i} } \right)} \right).$$

Eigenvector centrality of actor *i*:

4 $$C_{E} \left( {N_{i} } \right) = \lambda \sum\limits_{j}^{g} {x_{ij} } C_{E} \left( {N_{j} } \right),\;i \ne j.$$

### Topic modeling

Topic modeling is based on a statistical inference method that determines the probability of a word’s association with a certain topic, and the joint probability of the topic existing in a particular document. The LDA is one of the several statistical algorithms that can be used for topic modeling. It predicts the related words of a particular topic, based on the premise that documents with similar word distributions will contain similar topics (Blei et al., 2003). Blei et. al. (2003) introduced the LDA as the first approach that allows for modeling of the topic semantics entirely within the Bayesian statistical paradigm. According to their research, the aim of the LDA algorithm is to model a comprehensive representation of the corpus by inferring the latent content variables, called topics. Topics are heuristically located on an intermediate level between the corpus and the document that can be imagined as content-related categories, or clusters. A major advantage of topic modeling is that it requires no prior knowledge input to infer the topics from a given collection. Since topics are inferred and not explicit, no information about them is directly observable in the data.

**Fig. 1:**
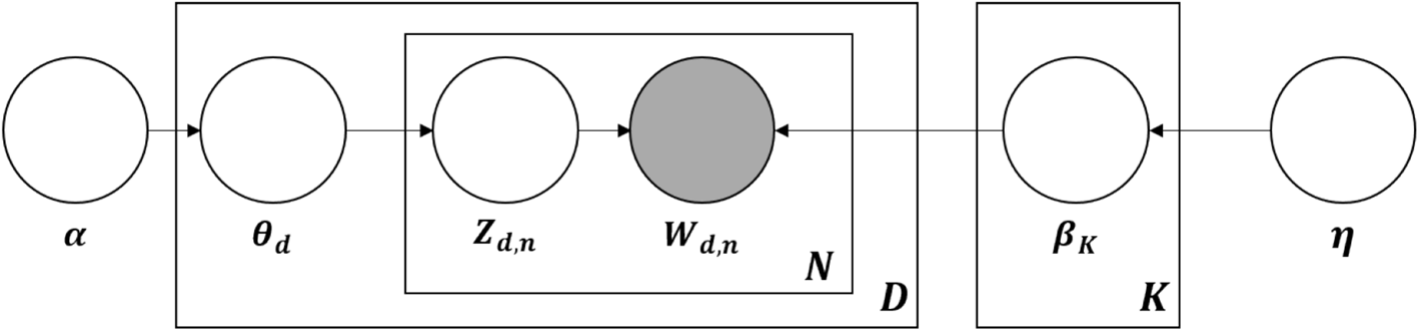
LDA model (Reprinted from Kim et al. (2016))

For topic modeling, we used the Gensim module provided by Python 3.7 and visualized the resulting data using the pyLDAvis module (Fig. 1), where: (1) $K$ represents the number of topics; (2) $\alpha$ is the value of the Dirichlet prior weight of topic *k* by document, and is a parameter that determines the value of $\theta$; (3) η is the Dirichlet prior weight of word *w* by topics, and is a parameter that determines the value $\beta$; (4) $\theta_{d}$ is the ratio of the topics per document; (5)$\beta_{k}$ is the probability of generating the word w by topic; (6) $Z_{d,n}$ is the topic of the *n*th word of the document *d*; and (7) $W_{d,n}$ is the *n*th word of document *d* (observed variable in the document; Kim et al., 2016).

### Sentiment analysis

Sentiment analysis is a text mining technique that extracts the key opinions, emotions, attitudes, and dispositions from a large amount of text data to estimate and classify the author’s emotions (Feldman, 2013; Lee et al., 2016). In general, the data are classified in a binary form as positive or negative and are further sub-categorized into multi-category sensibilities such as sadness, anger, or happiness. An emotional analysis is carried out through subjectivity detection and a polarity detection (Yeon et al., 2011). The text collected in the subjectivity detection stage is then classified into elements to be used for sentiment analysis after eliminating the unrelated terms. In the polarity detection phase, a sentiment dictionary is used to determine whether the given data is positive or negative. Next, the polarity detection is conducted using two methods: machine learning, and emotional dictionaries. Machine learning classifies the contents of a given document as positive or negative through patterns contained in the document; from these manually coded documents, the training data are then extracted, which are used to create machine-based models for determining the emotional valence of the document. Emotional classification analyzes the subjectivity of documents according to an emotional dictionary, which is created by recording the polarity of the predicates that are polar (adjectives) (Oh & Chae, 2015).

Using the above methods, we classified each tweet into positive, neutral, and negative emotions using WordNet, which is an English emotional dictionary provided by the Princeton University for sentiment analysis. From the crawled tweets, we extracted the tweets that mentioned fashion brand and used a positive and negative frequency analysis to determine the customers’ responses to each of the brands. Equation 5 presents the formula for the sentimental evaluation of a brand used in this study.

Formula for the sentimental evaluation of a brand:

5 $$Sentimental\;evaluation\;of\;a\;brand = \frac{Number\;of\;positive \;tweets \;of\;the\;brand}{{Total\;number\;of\;tweets\;of\;the\;brand}}.$$

## Results

### Semantic network analysis by city

Based on the frequency of appearance, the top 100 keywords, including “designers,” “brands,” “influencers,” “fashion items,” “designs,” “materials,” and “themes,” were extracted, classified, and visualized through the Clauset–Newman–Moore (CNM) algorithm. The macroscopic properties of each network are indicated in Table 2.

**Table 2:** Macro graph metrics of the top 100 keywords by cities

	Paris	Milan	New York	London
Total edges	2706	1390	4096	1904
Maximum geodesic distance	3	5	2	4
Average geodesic distance	1.742	2.084	1.5704	1.9012
Graph density	0.273	0.140	0.413	0.192
Average degree	27.060	27.800	81.920	38.080
Average betweenness centrality	37.630	107.300	58.040	91.120
Average degree closeness centrality	0.006	0.005	0.006	0.005
Average eigenvector centrality	0.010	0.010	0.010	0.010
Average clustering coefficient	0.522	0.431	0.624	0.461

### 2019 F/W Paris fashion week

**Fig. 2.**
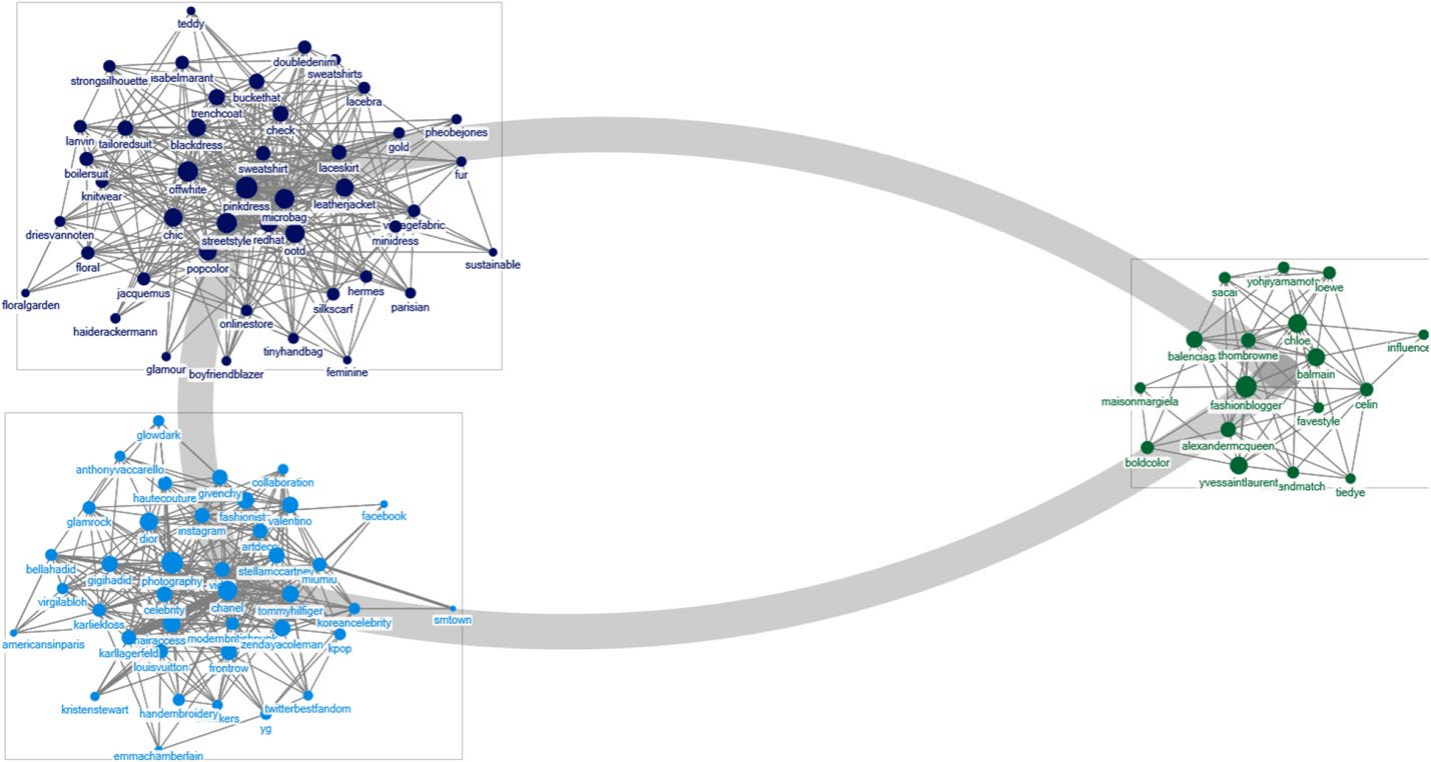
Classified groups of keyword clusters using CNM Algorithm: 2019 F/W Paris fashion week

A network analysis of Twitter for the 2019 F/W Paris Fashion Week reveals that “Chanel (814),” “Tommy Hilfiger (681),” “Saint Laurent (375),” “Zendaya Coleman (360),” “Dior (328),” “Gigi Hadid (253),” “Balmain (224),” “street fashion (217),” “Miu Miu (207),” and “Off-White (203)” were the most frequently mentioned keywords (Table 3). Using the CNM algorithm, the data was classified into the following groups: item groups, social networking sites (SNS) and influencers, brand and designer groups, design formats, and brand groups (Fig. 2). The item group included keywords such as “double denim,” “sweat shirt,” “lace bra,” “teddy bear coat,” “fur coat,” “lace skirt,” “leather jacket,” “micro bag,” “boyfriend blazer,” “trench coat,” “bucket hat,” and “boiler suit,” which were connected to the brand and the designer that launched the item. The terms related to SNS and influencers such as “Facebook,” “Instagram,” “Twitter,” and “celebrity” were categorized into the same group. Additionally, among the influencers, models “Gigi Hadid,” “Bella Hadid,” and the celebrities affiliated with YG and SM town were mentioned with the term “K-Pop.”

**Table 3:** Top ten keywords by frequency

Keyword	Frequency	${{\text{C}}_{\text{d}}}^{\text{a}}$	${{\text{C}}_{\text{b}}}^{\text{b}}$	${\text{C}_{\text{c}}}^{\text{c}}$	${\text{C}_{\text{e}}}^{\text{d}}$	C. Coef	Group
2019 F/W Paris fashion week							
Chanel	814	0.266	120.985	0.007	0.018	0.374	2
Tommy Hilfiger	681	0.195	70.643	0.006	0.013	0.382	2
Saint Laurent	375	0.221	76.256	0.006	0.016	0.402	3
Zendaya Coleman	360	0.201	73.857	0.006	0.014	0.376	2
Dior	328	0.236	92.752	0.007	0.017	0.431	2
Gigi Hadid	253	0.190	73.895	0.006	0.013	0.413	2
Balmain	224	0.226	85.474	0.007	0.016	0.417	3
Street style	217	0.291	165.474	0.007	0.020	0.373	1
Miu Miu	207	0.130	84.210	0.006	0.010	0.489	2
Off-white	203	0.296	158.081	0.007	0.020	0.379	1
2019 F/W Milan fashion week							
Gucci	277	0.381	823.79	0.006	0.024	0.246	2
Versace	275	0.472	923.264	0.007	0.029	0.256	3
Moschino	225	0.211	75.292	0.005	0.016	0.424	3
Prada	171	0.301	551.936	0.006	0.018	0.244	1
Street style	135	0.432	840.198	0.006	0.027	0.252	1
Giorgio Armani	121	0.180	150.412	0.005	0.011	0.275	3
Gigi Hadid	116	0.211	123.766	0.005	0.015	0.371	3
Karl Lagerfeld	115	0.241	135.926	0.006	0.018	0.362	1
OOTD	108	0.391	451.814	0.006	0.027	0.298	1
Maxmara	90	0.261	116.672	0.006	0.020	0.403	3
2019 F/W New York fashion week							
OOTD	878	0.964	476.278	0.01	0.02	0.411	2
Street style	836	0.793	287.232	0.008	0.017	0.451	2
Michael Kors	701	0.603	115.081	0.007	0.014	0.534	2
Sustainable	532	0.814	255.981	0.009	0.018	0.451	2
Stripe dress	514	0.864	268.483	0.009	0.019	0.461	1
Fashion blogger	508	0.723	209.678	0.008	0.016	0.487	2
Ralph Lauren	433	0.522	65.091	0.007	0.013	0.597	1
Marc Jacobs	356	0.492	58.444	0.007	0.012	0.584	1
Instagram	346	0.693	146.264	0.008	0.016	0.515	2
Faux fur coat	337	0.723	163.451	0.008	0.017	0.521	1
2019 F/W London fashion week							
Burberry	298	0.391	273.537	0.006	0.02	0.352	2
Victoria Beckham	295	0.432	473.376	0.006	0.023	0.315	1
Street style	225	0.472	458.639	0.007	0.024	0.33	2
Fashion blogger	159	0.582	608.618	0.007	0.028	0.296	2
Fashion scout	156	0.180	40.947	0.006	0.011	0.471	1
OOTD	141	0.502	450.563	0.007	0.025	0.313	3
Fuchsia pink dress	134	0.532	457.195	0.007	0.027	0.321	1
Positive fashion	129	0.291	251.198	0.006	0.011	0.273	1
Sustainable	127	0.301	187.315	0.006	0.015	0.34	2
Vivienne Westwood	120	0.251	75.324	0.006	0.014	0.44	2

^a^Degree centrality

^b^Betweenness centrality

^c^Closeness centrality

^d^Eigenvector centrality

Words pertaining to fashion bloggers, such as “fave style,” “fashion blogger,” and “mix and match,” also emerged as keywords even though these terms are not directly related to the fashion week. These keywords emerged because of the fashion bloggers’ repeated reference to the fashion trends and styles presented in the fashion week, which reflects the people’s attention toward the apparel worn in the fashion week. In particular, for terms such as “photograph” or “fashion blogger,” the connection and the eigenvector centers showed higher values than “brands,” “designers,” “celebrities,” and certain fashion items. An eigenvector centrality represents the degree of influential keywords possessed by a node. Therefore, we infer that external factors such as fashion bloggers and their social media activities are influential in the fashion week.

Based on connection-centricity, “Off-White,” “Chanel,” and “Chloe” are the most influential brands. Among fashion items, “micro bag,” “leather jacket,” and “black dress” received the most attention. Important themes and styles associated with the collections presented by the brands are “art deco,” “glam rock,” “sustainable,” and “feminine.” Furthermore, “Parisian” and “Haute Couture” emerged as unique keywords related to the Paris Fashion Week. Unlike the results from Milan, London, and New York, the top keywords mentioned in Paris Fashion Week in Paris were mostly related to fashion brands rather than influencers. Consistent with the claim presented by Joo (2016), the trends from the Paris Fashion Week indicates that Paris is a traditional fashion city that possesses artistic, multicultural, technical, and industrial distinctions based on the French culture.

### 2019 F/W Milan fashion week

**Fig. 3.**
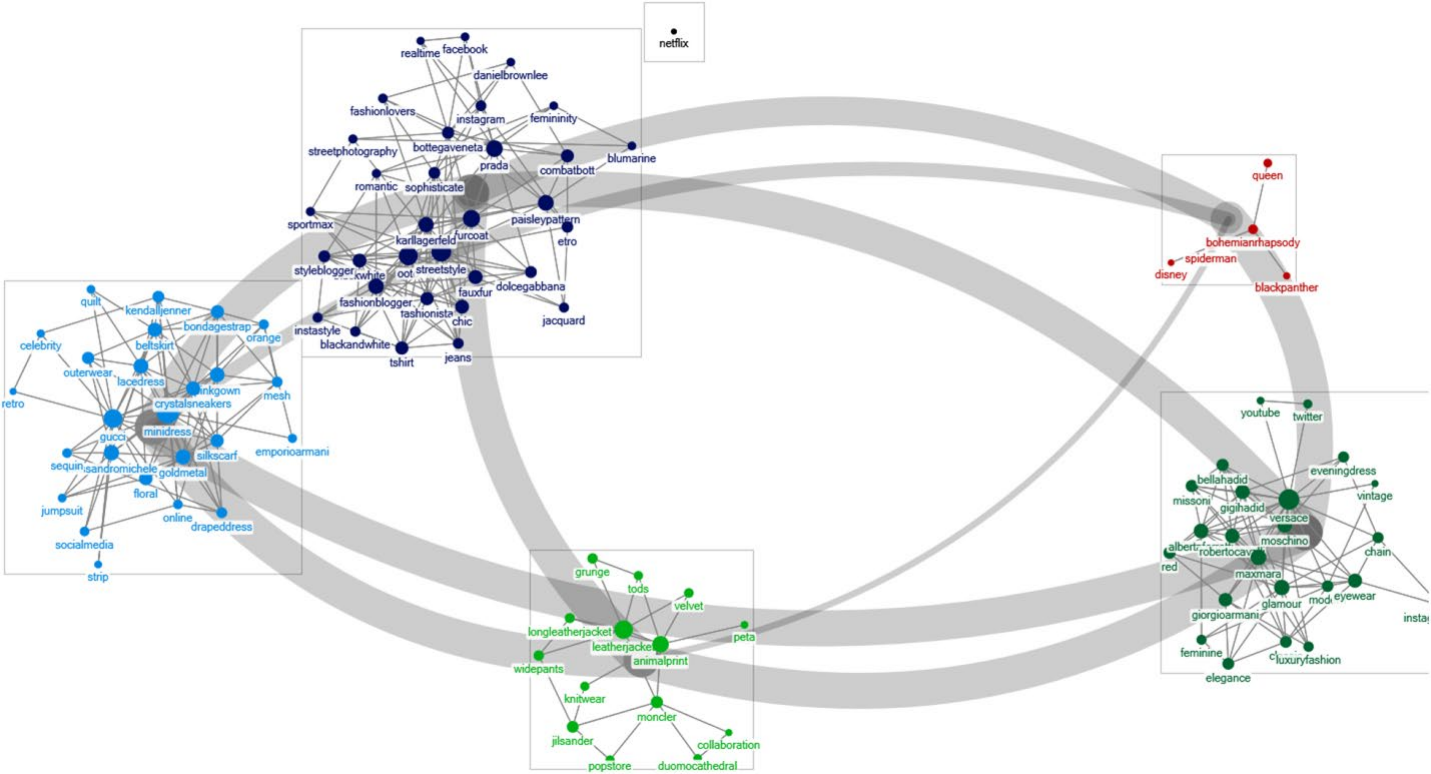
Classified groups of keyword clusters using CNM Algorithm: 2019 F/W Milan fashion week

A network analysis of the 2019 F/W Milan Fashion Week showed that “Gucci (277),” “Versace (275),” “Moschino (225),” “Prada (171),” “street fashion (135),” “Giorgio Armani (121),” “Gigi Hadid (116),” “Karl Lagerfeld (115),” “OOTD (outfit of the day; 108),” and “Max Mara (90)” were the most frequently-appearing keywords (Table 3). A classification using the CNM algorithm revealed six groups and three major clusters (Fig. 3). Frequently cited words showed similar degree, closeness, betweenness, and eigenvector centralities. The clusters with the most nodes contained references to SNS/fashion bloggers, fashion design sensibilities and elements, and major brands. Interestingly, keywords such as “real-time,” “Facebook,” and “Instagram” all showed connectivity, proving that SNS enabled a rapid proliferation of video and postings about the fashion week.

Compared to the tweets from other three cities, tweets from the Milan Fashion Week featured more references to the materials and patterns used in the clothes produced, such as “quilt,” “mesh,” “metal,” “sequin,” “velvet,” “leather,” “floral,” and “stripes.” Furthermore, words related to diverse fashion items frequently recurred. According to Joo (2016), the Milan Fashion Week tends to feature collections that combine both New York's practicality and Paris’ creativity. Therefore, the frequently-mentioned keywords are in line with the practical and the creative aspects of the New York and the Paris collections, respectively. In addition, faux (artificial) fur showed connectivity with the animal protection organization People for the Ethical Treatment of Animals, which was also associated with “animal print.” The results indicate that ethical fashion is becoming a hot topic among fashion brands and designers in Milan.

Three groups contained references to fashion brands and fashion items. Fashion items that were mentioned include “gown dress,” “silk scarf,” “jumpsuit,” “mini dress,” “wide pants,” and “leather jacket,” and related trends such as “grunge,” “retro,” “vintage,” and “glam” also appeared. The emergence of distribution channels such as “online stores” and “pop stores” indicated public interest in popular items presented in the collection. Words not directly related to the collections, such as “Bohemian Rhapsody,” “Queen,” “Spider Man,” “Disney,” “Black Panther,” and “Netflix” appeared in relation to the Oscars Awards due to the celebrities and models who attended the fashion week.

Based on connection-centricity, Versace, Gucci, and Prada are the most influential brands in the Milan Fashion Week, and “mini dress,” “leather jacket,” and “fur coat” were the fashion items that attracted the most attention. These fashion items were closely related to the theme and the sentiment of the respective brands that presented the items. The term “Duomo cathedral” appeared both as the place where the collection was displayed, and as a keyword reflecting the characteristics of the city.

### 2019 F/W New York fashion week

**Fig. 4.**
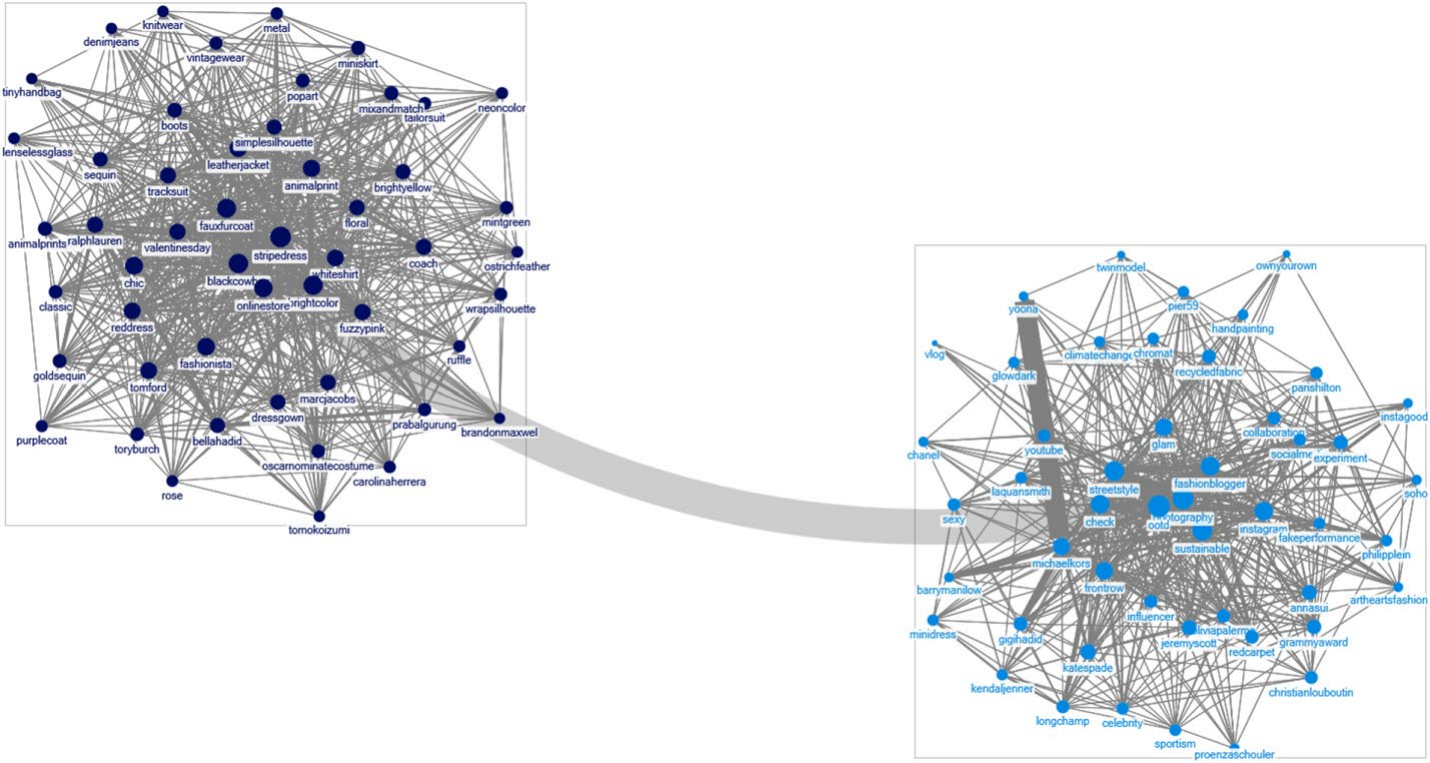
Classified groups of keyword clusters using CNM Algorithm: 2019 F/W New York fashion week

A network analysis of the 2019 F/W New York Fashion Week reveals that “OOTD” (878), “street fashion” (838), “Michael Kors” (701), “sustainable” (532), “stripe dress” (514), “fashion blogger” (508), “Ralph Lauren” (433), “Marc Jacobs” (356), “Instagram” (346), and “faux fur” (337) were the most frequently cited words (Table 3). These keywords were then classified into two groups based on the number of nodes using the CNM algorithm (Fig. 4). SNS and celebrities appeared in the same group, whereas other keywords related to design features, items, and brands were spread across different groups.

Olivia Palermo, Kendal Jenner, Gigi Hadid, Barry Manilow, Paris Hilton, and Yoona, and were some of the prominent celebrities/models that appeared in the same group, who were also related to words such as “red carpet” and the “Grammy award.” While fashion bloggers were considerably influential at the Milan Fashion Week, celebrities and models who appeared in the New York Fashion Week were actively mentioned on Twitter with words related to the Grammy Awards. Social media-related keywords appeared in the same group as the celebrities, and “Soho” appeared as a keyword that reflected regional characteristics.

The New York Fashion Week is the largest fashion week. It features popular trends and primarily focuses on practicality rather than innovation, differentiating it from the Paris Fashion Week, which focuses on art and design; Milan, which presents work based on the textile industry; and London, which features innovative designs (Joo, 2016). Nevertheless, despite being known for its traditional and commercial designs, the New York Fashion Week featured the highest number of ethical fashion-related keywords among the four cities: “sustainable,” “faux fur,” “climate change,” and “recycled fabric.” This phenomenon suggests that fashion trends in New York have shifted their focus from practical to ethical fashion.

Based on connection-centeredness, fashion brands such as “Michael Kors,” “Tom Ford,” and “Ralph Lauren” are the most influential brands, and “stripe dresses,” “faux coats,” and “leather jackets” were the most popular fashion items. The New York Fashion Week was also associated with style themes such as “chic,” “glam,” and “experimental,” which showed the overall sentiment of the New York collection. Furthermore, among the four fashion weeks, the clothing collections in New York had the most diverse range of colors despite being held in the F/W season, words associated with light and saturated colors, such as “bright color,” “bright yellow,” “fuzzy pink,” “mint green,” and “neon color” frequently appeared. “Simple silhouette” and “wrapped silhouette” were the most commonly cited silhouettes. White shirts and check patterns were the most common in terms of their betweenness centrality, making them the most used designs feature in the New York F/W collection.

### 2019 F/W London fashion week

**Fig. 5.**
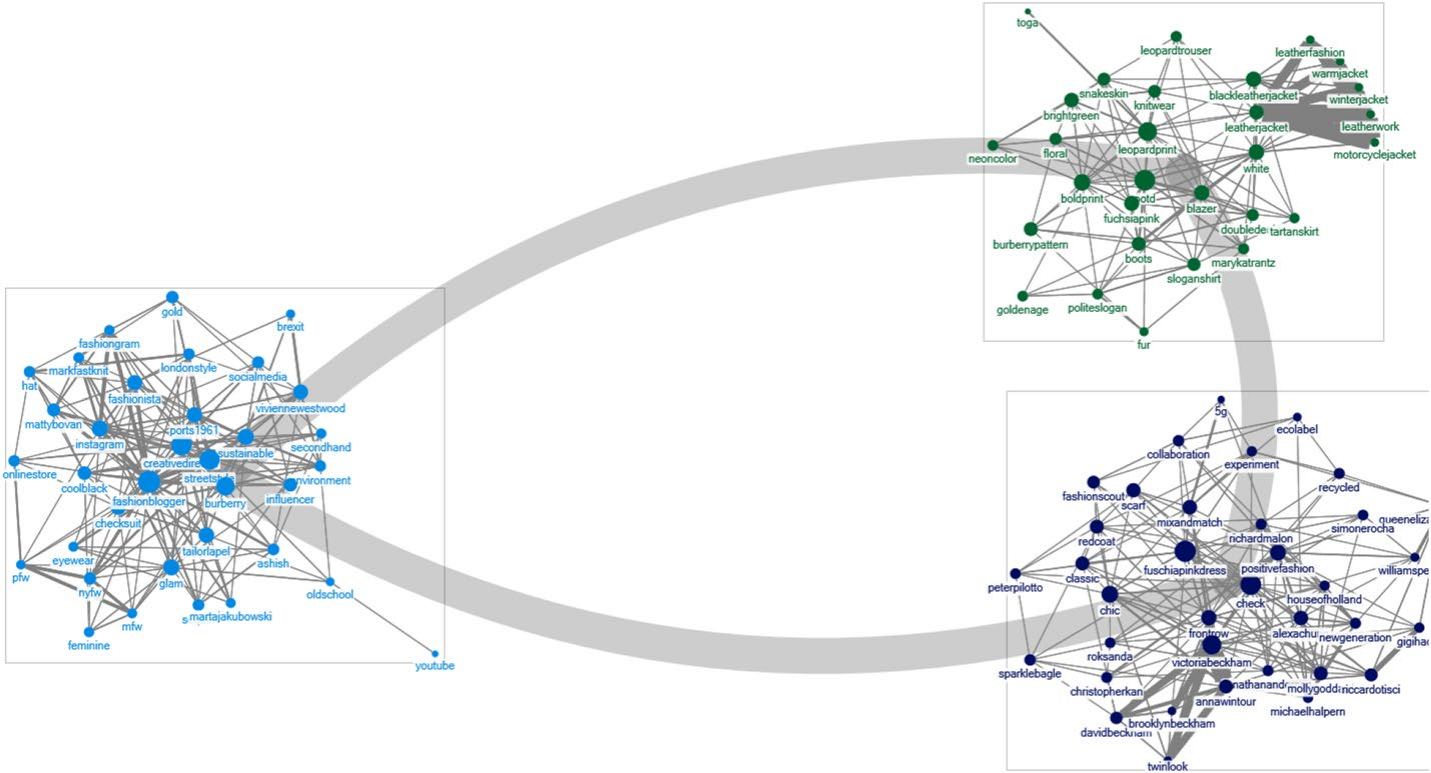
Classified groups of keyword clusters using CNM Algorithm: 2019 F/W London fashion week

Table 3 shows the keywords from a network analysis of Twitter for the 2019 F/W London Fashion Week in the order of their frequencies. The data were classified into three groups using the CNM algorithm. The SNS/influencer group and the design element group were differentiated in distinct groups whereas keywords pertaining to brands, ethical fashion, and celebrities were distributed among three groups (Fig. 5).

“Instagram,” “YouTube,” “fashion-gram,” “influencer,” “fashion blogger,” and “fashionista” co-occurred in the same group, and terms related to outerwear, such as “leather jacket,” “motorcycle jacket,” “warm jacket,” “winter jacket,” and “blazer” recurred in the item/design group. Design elements such as “motorcycle jacket,” “warm jacket,” “winter jacket,” and “blazer” also co-occurred.

Well-known figures who are not associated with the fashion industry also appeared: soccer player “David Beckham” was mentioned on Twitter as the husband of designer Victoria Beckham, and the British royal household members such as “Queen Elizabeth” and “William Spencer” also appeared. Political keywords such as “Brexit” were also mentioned, as some designers criticized Brexit through their collections. For instance, Vivienne Westwood used her designs to disparage global climate change, Brexit, and fast fashion.

Most keywords that appeared in London Fashion Week are related to the British fashion brands and designers. The degree centrality revealed “Victoria Beckham,” “Burberry,” “Ports 1961,” and “Vivienne Westwood” as the most influential keywords. Terms such as “chic,” “glam,” and “classic” were frequently referenced, and the term “old school” also appeared. Frequently used words also showed high results in degree, betweenness, closeness, and eigenvector centralities.

On Twitter, the London Fashion Week was associated with two keywords with opposing concepts. Keywords related to ethical fashion such as “sustainable,” “environment,” “ecolabel,” “recycled,” “secondhand,” and “positive fashion” also coexisted with animal-based materials such as “leopard,” “snake skin,” “fur,” and clothes with “animal prints.” In particular, the words “positive fashion” and “sustainable” had high betweenness centralities relative to their frequencies, indicating that brands, designers, and items are often referenced in relation to one another.

### Topic modeling in 2019 F/W fashion week

To construct a model with sufficient number of topics, we increased the number of topics from two to 50 in increments of 10, and thereafter created a model that increased in increments of one in the interval two to 20. Finally, three topics were selected for each city. Based on articles from Marie Claire (https://www.marieclaire.com) and Vogue’s websites (https://www.vogue.com/fashion-shows) that have free access to information about fashion shows, we conducted a sentiment analysis to investigate the degree to which the results matched the topics. The results showed similar design inspirations, collection themes, and brands for the same topic. The topics related to people such as the Beckham couple or the Hadid sisters primarily focused on the relationship between people.

### F/W Paris fashion week

**Fig. 6.**
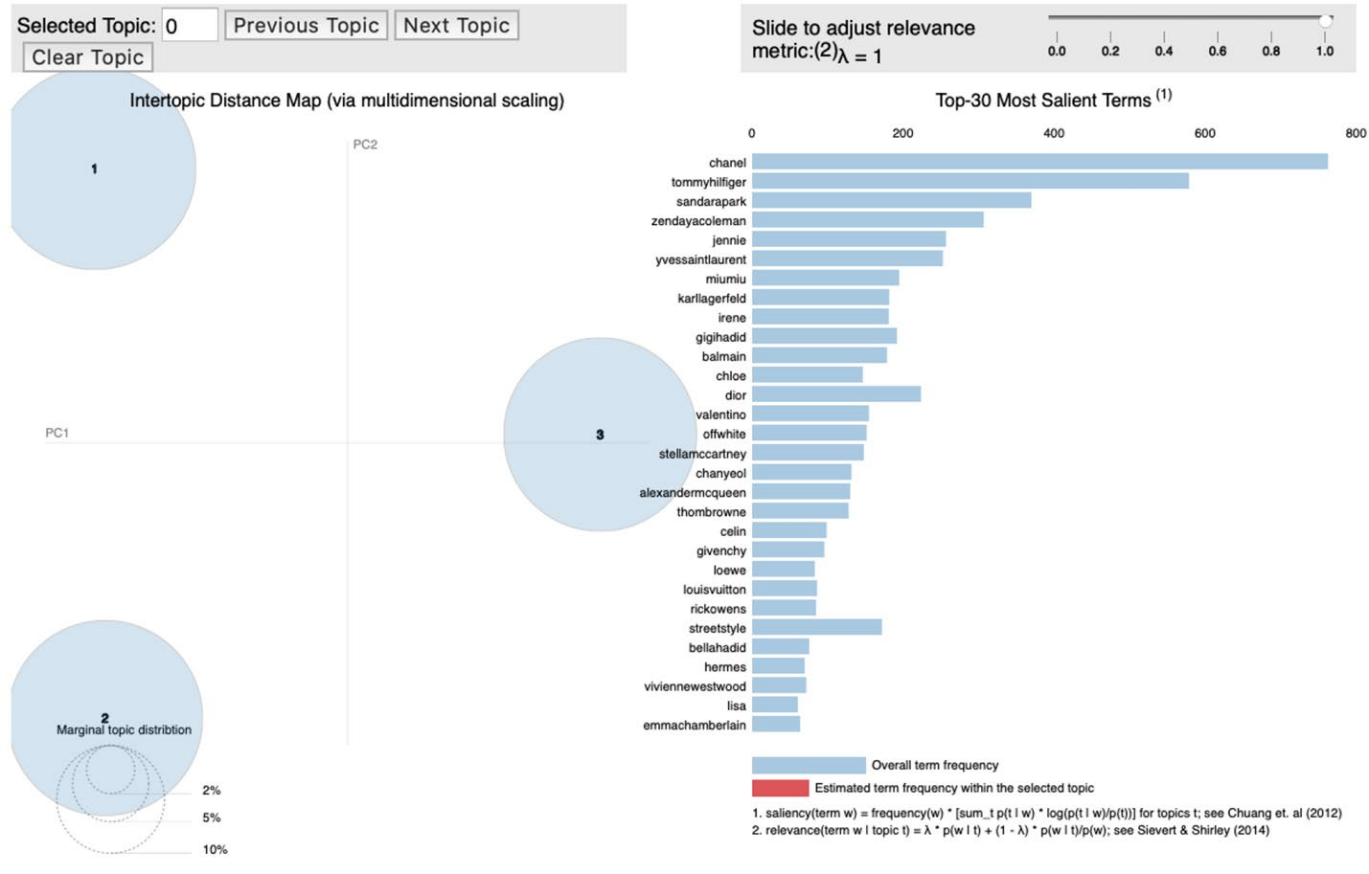
Topic modeling: 2019 F/W Paris fashion week

For the 2019 Paris F/W collection, the following brands had the highest probability of distribution in Topic 1: “Balmain,” “Valentino,” “Off-White.” “Stella McCartney,” “Alexander McQueen.” In Topic 2, “Tommy Now,” “Miu Miu,” “Dior,” “Givenchy,” and “Hermes” appeared, and “Chanel,” “Saint Laurent,” “Chloe,” “Celine,” and “Loewe” had the highest probability of distribution in Topic 3 (Fig. 6).

Topic 1 brands had well-tailored jackets, angled shoulder lines, and funky ambiences as their prominent features, and were characterized as “tailored street style.” Balmain featured a well-tailored uniform jacket, crisp ruffled dress, masculine shoes, and leather pants similar to those worn by pop stars in the 1980s. Valentino presented a funky-style floral print, neon color silk dress, and clothes exhibiting a street mood, while Off-White featured optical patterns, metallic trench coats, jersey dresses with asymmetrical hemlines, and primary color leather coats that centered around street wear and checkerboard prints.

Brand collections under Topic 2 mainly featured clothes athleisure style as exemplified by the brand Tommy Now, which depicted a sporty mood based on supermodels from the 1980s and 1990s and disco queens from the 1970s. Miu Miu exhibited a millennial sentiment expressed through long capes, mini dresses with ruffle decorations, chunky boots, and big bags. Dior combined a tailored jacket having a masculine silhouette with a big skirt, a new look silhouette dress, a sporty jumper, kitten heels, a bucket hat, and cropped suit pants. Brands under topic four exhibited clothes with classic and feminine moods. For instance, Chanel, the most exemplary brand in Topic 3, featured a tweed houndstooth check suit, a candy-colored sweater with a classic Chanel logo, and a feather-detail cocktail dress. The large shoulder line, straight silhouette, and polka-dot tights made the tuxedoes architectural and feminine.

### F/W Milan fashion week

**Fig. 7.**
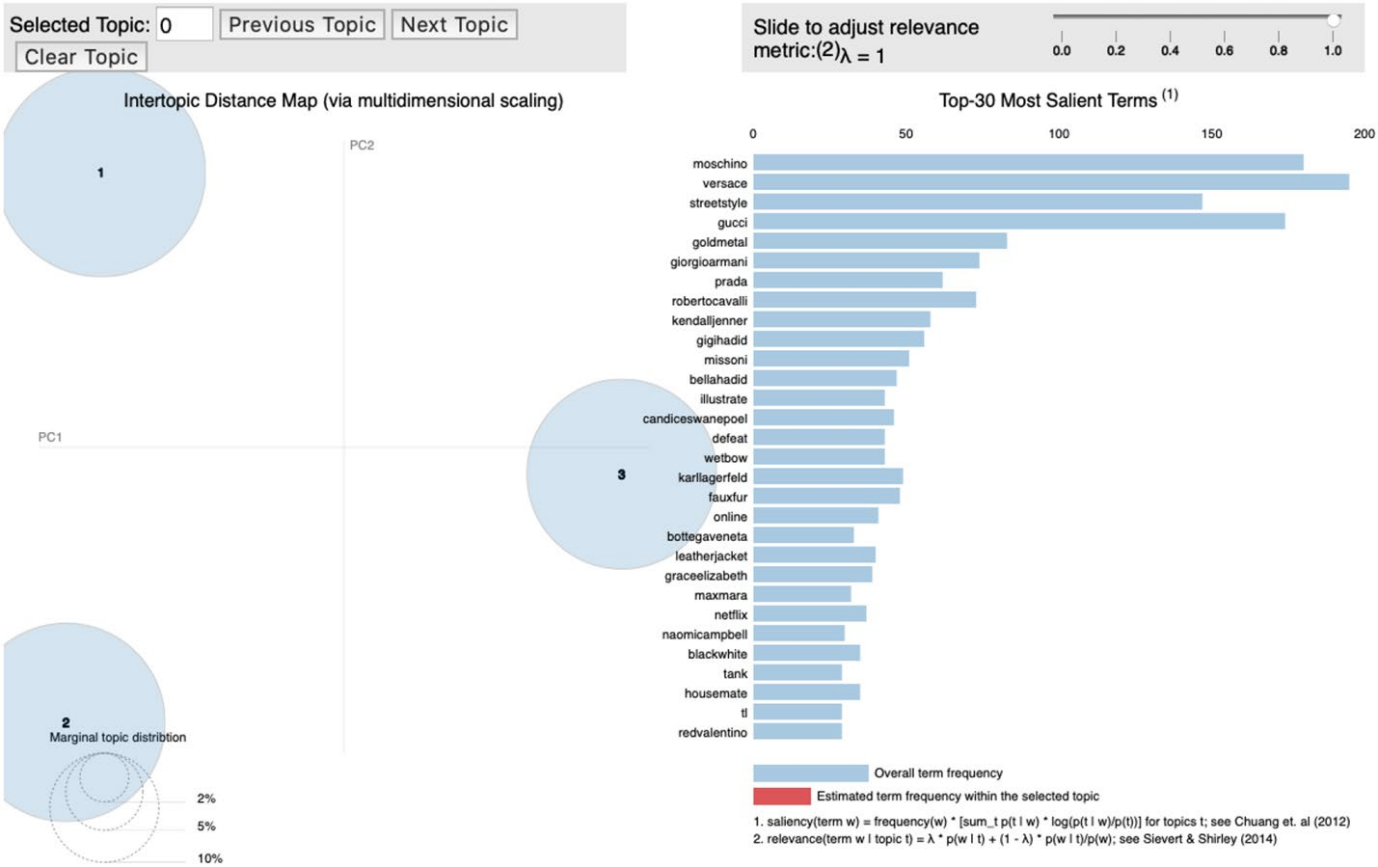
Topic modeling: 2019 F/W Milan fashion week

For the 2019 Milan F/W collection, “Giorgio Armani,” “Roberto Carvalli,” “Gucci,” “Dolce Gabbana,” and “Alberta Ferretti,” had the highest probability of distribution for Topic 1, whereas Topic 2 comprised of brands such as “Versace,” “Moschino,” “Bottega Veneta,” and models “Gigi Hadid,” “Bella Hadid,” and “Candice Swanepoel” (Fig. 7).

Topic 1 was named “tailored” because the most representative brand in Topic 1, Giorgio Armani, featured clothing with dark and calm colors, complex tailoring, and luxurious materials such as silk, velvet, and leather. Other major brands in Topic 1 also presented clothing that aligned with this theme. For instance, Roberto Carvalli also had elegantly tailored coats, classic neckline dresses, boots that delicately covered people’s legs, and geometric patterns and design. Gucci also displayed colorful genderless designs based on complex tailoring.

Topic 2 was labelled “Retro” the brands featured under this topic expressed a vintage sentiment popular during the 1970s, 1980s, and 1990s. Moschino staged kitsch gold jewelry matched with voluminous retro hair prevalent in the 1970s, along with various monetary patterns, cartoon characters, and humorous baby bear prints. Versace exhibited a grunge look from the 1990s consisting of old-fashioned cut-off detail, messy hair, retro-style bustiers; gorgeous colors like gold, yellow, blue, pink and orange, and utilized silky materials, delicate laces, classic patterns, glam, luxury moods, slip dresses and jackets.

Brands under Topic 3 developed genderless designs featuring masculine and linear silhouettes, and was therefore labelled “Genderless.” Gucci appeared in all the topics due to its elaborate tailoring (Topic 1) retro mood (Topic 2), and genderless silhouettes (Topic 3). Prada harmoniously combined romantic details such as flowers, lace, and ribbons with a military mood with masculine pants, and a goth mood. Missoni expressed a genderless design through classic and geometric patterns and large neck decorations. Max Mara utilized beige, camel, and navy-focused color palettes, a maximal coat, multiple-layered jackets, and a professional-looking suit.

### F/W New York fashion week

**Fig. 8.**
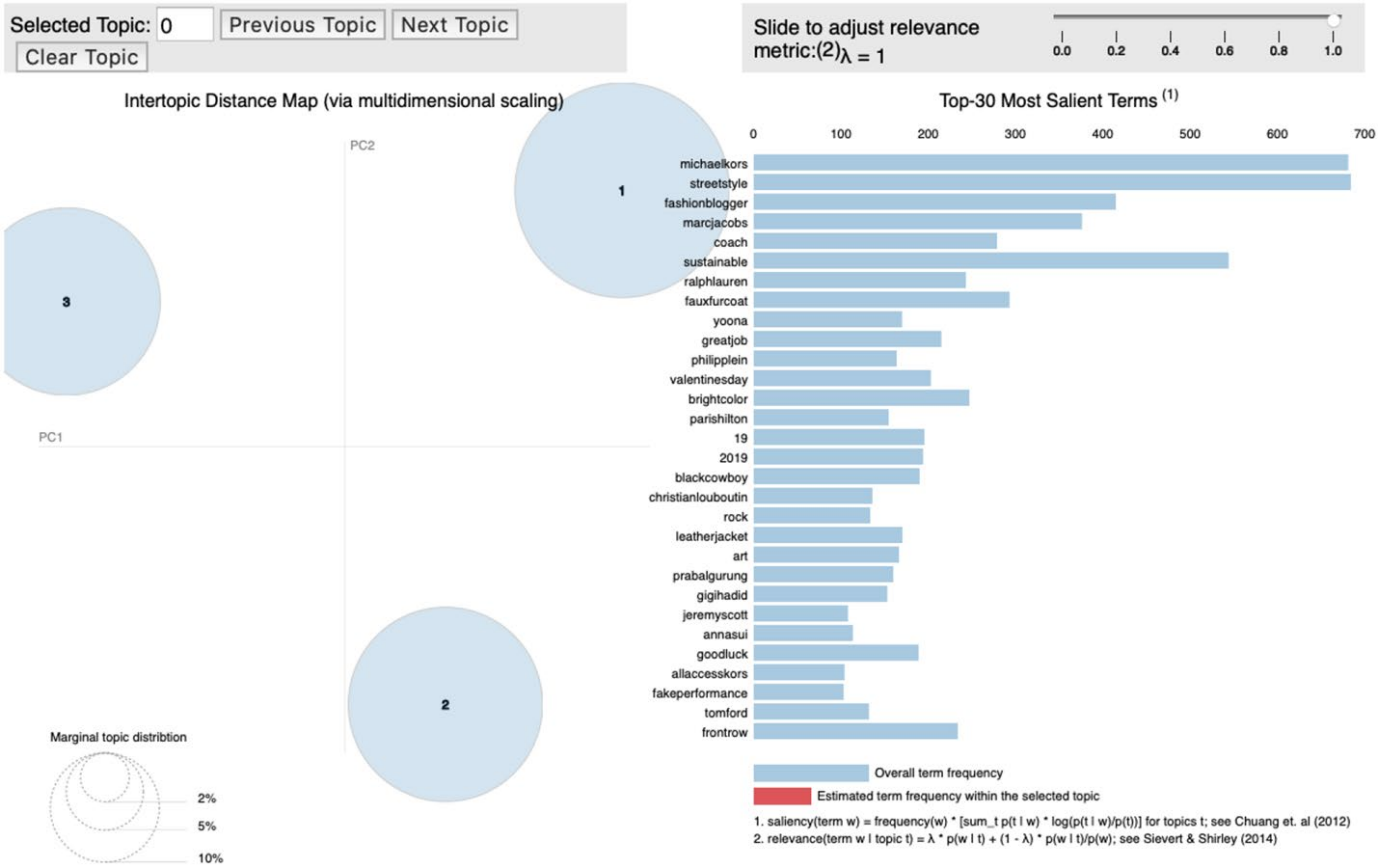
Topic modeling: 2019 F/W New York fashion week

Based on the data collected from the New York Fashion Week, brands such as “Marc Jacobs,” “Prabal Gurung,” “Tom Ford,” and “Tory Burch” were placed under Topic 1. Topic 2 was mostly comprised of words regarding social media influencers, such as “fashion blogger,” “photography,” “OOTD,” “front-row,” “fashionista,” “social media,” and “celebrity,” rather than brand names, excluding “Ralph Lauren.” Topic 3 featured brands such as “Michael Kors,” “Coach 1941,” “Jeremy Scott,” and “Longchamp” (Fig. 8).

Topic 1 was labelled “Classic and romantic” because it was characterized by various design details, fashion items, and classic and romantic moods. Marc Jacobs’ collection had rich silhouettes, a classic cape and coat, a couture dress decorated with artificial feathers and flowers, and a feather dress. Prabal Gurung presented a look that displayed an oriental style, contrasting color matches reminiscent of Yves Saint Laurent and Christian Lacroix, ethnic jacquard fabrics, bold fringes, and oriental embroidery. Tom Ford expressed sensuality through a pale blue shirt, purple satin pants, silk jersey, and a finale dress paired with a bold chain. Tory Burch presented classic, tailored coats and trousers, masculine silhouettes, floral patterns, and ruffles, along with pleated romantic blouses and skirts.

Ralph Lauren, which appeared under Topic 2, presented an elegant and dramatic look that displayed a military mood, a sailor look, and embodied American luxury through a color palette consisting of black, gold and white. However, Topic 2 mostly comprised of keywords such as “fashion blogger,” “photography,” “OOTD,” “front-row,” “fashionista,” “social media,” and “celebrity.” Therefore, Topic 2 was labelled “influencer.”

The common characteristics of the brands shown in Topic 3 were a street mood with a 1970s and 1980s vibe, and leather jackets. Topic 3 was therefore named “Vintage and Retro.” Michael Kors portrayed designs popular during the 1970s through a floral dress that featured a shearing fur stole, fringes, beads, a slip dress with feathers, a check pants suit, Boeing glasses, and a newsboy cap. In contrast, Coach 1941, displayed a bohemian mood with a psychedelic quilted pattern, a floral and a marbling pattern reminiscent of geological layers, classic tartan checks, and Bermuda pants. Jeremy Scott utilized black and white colors, exaggerated accessories such as a gigantic ribbon, an A-line tulle, a rider jacket, and a hoodie to convey a 1970s and 1980s sentiment. Furthermore, Longchamp expressed a similar sentiment through its rock-style leather clothing, animal prints, ethnic patterns, signature logo patterns, and a miniature-sized white Le Pliage bag.

### F/W London fashion week

**Fig. 9.**
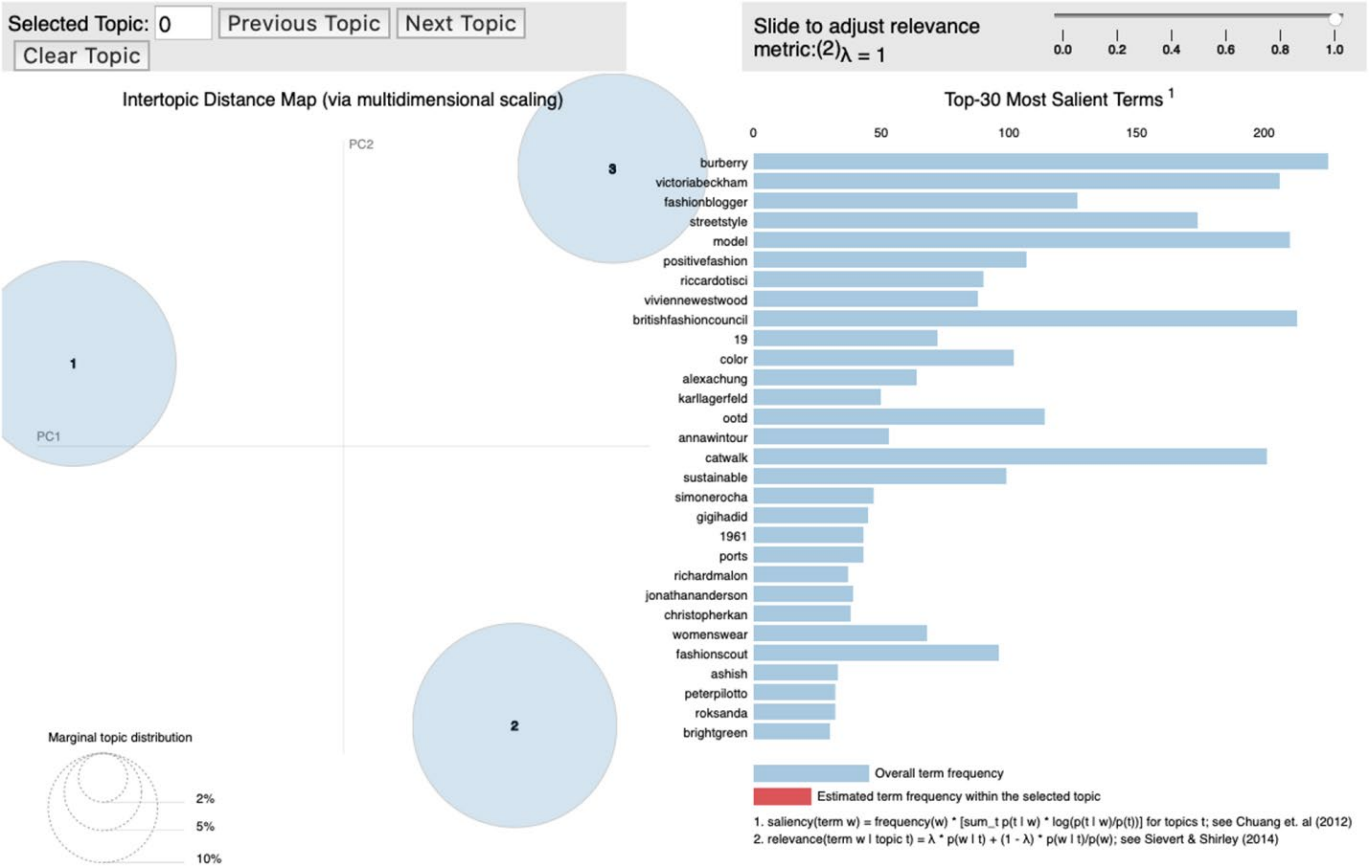
Topic modeling: 2019 F/W London fashion week

The data from the 2019 London Fashion Week indicated that brands such as “Victoria Beckham,” “Simone Rocha,” “J.W. Anderson,” “Peter Pilotto,” “Roksanda,” and “Christopher Kane” were most associated with Topic 1. Topic 2 included keywords such as “Burberry,” “Vivienne Westwood,” “Alexa Chung,” “Ports 1961,” “Ashish,” and “Mary Katrantzou,” and Topic 3 featured the words “Richard Malone,” “sustainable,” and “recycled.” The brands that appeared in Topic 1 expressed a modern and feminine businesswoman, or displayed a romantic look frequently associated with young girls. Therefore, Topic 1 was named “Businesswoman and Romantic.” Victoria Beckham presented a businesswoman look through refined beauty, checkered pencil skirts, flare pants, and silk shirts. Furthermore, her look featured smooth and feminine colors such as lilac, turquoise blue and red. A cape-shaped manic silhouette coat with a pointed long collar and a wide banding at the waist portrayed a modern and feminine sensibility. Simone Rocha harmoniously combined an abstract and a bizarre sentiment conveyed through swirly patterns and rich balloon silhouette dresses with cobwebs with an opposing sense of romanticism. J.W. Anderson presented a voluminous dress in colorful chiffon, a flat patterned jacket, and oversized choker chains and belts (Fig. 9).

In Topic 2, brands exhibited a combination of various themes and asymmetrical silhouettes. Therefore, Topic 2 was named “Heterogeneous and Unbalanced.” Burberry, a brand that had the highest probability of being included in Topic 2, presented two distinct concepts. In the boys and girls section, it presented a street look with sportswear, lingerie dresses, and track pants, whereas in the Gentleman and Lady section, it included formal and classic items such as trench coats and tailored jackets to express the coexistence of classical and punk styles. Vivienne Westwood unveiled its signature wool coats with signature check patterns, painted-print leggings and jackets, and satin dresses, along with messages criticizing Brexit, global climate change, and fast fashion. Alexa Chung featured silhouettes that were popular during the 1940s through ruffles and glossy materials such as velvet and leather with floral patterns. In Topic 3, Richard Malone was the only fashion house that was included, and the topic was named “Sustainability” because it appeared with words such as “sustainable” and “recycled.” Accordingly, Richard Malone’s collection used organic cotton and recyclable materials that were sustainable and experimental (Table 4).

**Table 4:** Classification of themes in the 2019 F/W Fashion Week collections by cities

Cities	Topic number	Collection theme	Examples of fashion brand
Paris	Topic 1	Tailoring and street style	e.g., Balmain, Valentino, Off-White
	Topic 2	Athleisure style	e.g., Tommy Now, Miu Miu, Givenchy
	Topic 3	Classic and feminine	e.g., Chanel, Chloe, Celin
Milan	Topic 1	Tailored	e.g., Giorgio Armani, Roberto Carvalli, Dolce & Gabbana
	Topic 2	Retro	e.g., Moschino, Versace, Gucci
	Topic 3	Genderless	e.g., Prada, Missoni, Max Mara
New York	Topic 1	Classic and romantic	e.g., Marc Jacobs, Prabal Gurung, Tom Ford, Tory Burch
	Topic 3	Vintage and retro	e.g., Michael Kors, Coach 1941, Jeremy Scott, Longchamp
London	Topic 1	Businesswoman and romantic	e.g., Victoria Beckham, Simone Rocha, J.W. Anderson, Peter Pilotto, Roksanda
	Topic 2	Heterogeneous and unbalanced	e.g., Burberry, Vivienne Westwood, Ports 1961

### Sentiment analysis in 2019 F/W fashion week by brands

To perform a sentiment analysis of the Big 4 Fashion Weeks, we first extracted the nouns from the tweets crawled from Twitter, through stemming and listed them by word frequency. Thereafter, we categorized the three of the most frequently-mentioned brands for each city (shown in Table 1) as follows: Paris Fashion Week—Chanel (814), Dior (328), and Balmain (224); Milan Fashion Week—Gucci (277), Versace (275), and Prada (171); New York Fashion Week—Michael Kors (701), Ralph Lauren (433) and Marc Jacobs (356); and London Fashion Week—Burberry (298), Victoria Beckham (295), and Vivienne Westwood (120) (Table 5).

**Table 5:** Top three fashion brands by frequency in the 2019 F/W fashion weeks

	Paris	Milan	New York	London
Brand 1	Chanel	Gucci	Michael Kors	Burberry
Brand 2	Dior	Versace	Ralph Lauren	Victoria Beckham
Brand 3	Balmain	Prada	Marc Jacobs	Vivienne Westwood

The positive tweets pertaining to brand names extracted referenced brands such as Chanel (293), Dior (154), and Balmain (91) related to the Paris Fashion Week; Gucci (111), Versace (99) and Prada (73) appeared in tweets related to the Milan Fashion Week; Michael Kors (455), Ralph Lauren (190), and Marc Jacobs (138) appeared in tweets related to the New York Fashion Week; and Burberry (116), Victoria Beckham (148) and Vivienne Westwood (37) related to the London Fashion Week.

The results of the analysis indicate how these brands induced positive reactions from Twitter users (Table 6). The positiveness of a brand’s tweet’s was calculated through the percentage of positive tweets among the total number of tweets, calculated using Eq. 5. The resulting number is a quantitative representation of the sentimental evaluation of the brand. The top-ranked brands mentioned also had different positive rates depending on the people’s reactions. The most popular item calculated was the “Monogram handbag” from Michael Kors’ New York Fashion Week collection.

**Table 6:** Positive figures of top three brands in 2019 F/W fashion week’s ready-to-wear collection

	Paris		Milan		New York		London	
Brand 1	Chanel	0.36	Gucci	0.40	Michael Kors	0.65	Burberry	0.39
Brand 2	Dior	0.47	Versace	0.36	Ralph Lauren	0.44	Victoria Beckham	0.50
Brand 3	Balmain	0.41	Prada	0.43	Marc Jacobs	0.39	Vivienne Westwood	0.31

Note: The numbers represent the sentimental evaluation of the brand; Sentimental evaluation of the brand = Number of the positive tweets about the brand/total number of tweets about the brand

## Discussion

The present study used three methods to analyze tweets from the 2019 Paris, Milan, New York, and London Fashion Weeks: the social network analysis, which is a method of determining an entire network’s structure based on the relationship between its actors; topical modeling, a statistical analysis method that determines abstract topics contained in groups of texts; and sentiment analysis. A keyword analysis revealed the characteristics of the collections displayed and the attention received by certain celebrities and influencers. Topic analysis indicated the similarities between collections presented by fashion houses within the same topic. A sentiment analysis through WordNet showed the emotional valence of brands that were most frequently mentioned by customers.

The study has three main findings, outlined below. First, a semantic network analysis of the Big 4 Fashion Weeks’ Twitter data indicates that SNS and influencers were referenced more often than brands or designers. The street mood was the most commonly represented fashion sentiment, and leather jackets and faux fur jackets were the most popular fashion items. Interestingly, the textual data from all four cities, especially New York, contained words related to ethical fashion. These results reflect a change in the fashion industry, which had in the past been criticized for causing environmental pollution and animal abuse. The results also show a movement from commercial and practical designs, which were traditionally common in New York’s fashion industry, toward more sustainable designs. Second, a topic modeling based on tweets of Twitter users revealed that the data was best understood when classified into three topics for each city. In addition, most topics were classified based on the sentiment of the collection developed by the brand. Third, to analyze the sentiment of each brand, we used a sentiment analysis divided into three categories (positive, neutral, and negative). It revealed that even the brands that were frequently mentioned in Twitter showed differences in their positive ratings. The most acclaimed brand was Michael Kors from New York Fashion Week, and the fashion item that received the most public attention was the Monogram handbag.

The Paris Fashion Week has developed as a national brand that is contributing to building a positive national image, with support from the French government. In contrast, the New York Fashion Week receives sponsorships from corporations and the New York city. It has also devoted significant resources to marketing, thereby making the New York Fashion Week very corporate and bureaucratic. The Milan Fashion Week is significantly closed off to new and foreign designers and selects designers through internal examinations of their creativity, public relations, sales, and distribution rates. Additionally, several designers who presented their collections during the London Fashion Week left London due to low profitability. Most designers from London have their own small brands; however, some of them work for big fashion houses abroad (Joo, 2016). Our study reveals that each city’s fashion week reflects the city’s characteristics. For instance, Paris shows diversity in brands and designer spaces while New York generates popularity by appealing to people’s practical sensibilities.

The public’s interest in celebrities and influencers is a key finding that has various marketing implications. People usually express their desire to become like their role-models by imitating their fashion styles (Lee & Kim, 2019). Therefore, celebrities and influencers often create new fashion trends and increase the consumers’ desire to purchase new clothing styles. Our research confirms the influence exerted by various fashion bloggers and social media influencers by the frequency of tweets mentioning them. In fact, except in Paris, these influencers had more influence than brands or designers did. The results, therefore, indicate that fashion brands and designers can popularize their products by leveraging social media influencers. Currently, many fashion brands invite celebrities to advertise their products. However, they should go a step further and analyze the influence of influencers and models on social media to utilize their popularity for marketing purposes.

### Theoretical contributions

This study added meaningful insights to the fashion communication literature through an empirical analysis of the Big 4 fashion weeks. This study’s use of text mining, semantic network analysis, topic modeling, and sentiment analysis for quantitatively analyzing the keywords from the social media data about the fashion weeks deepened the discipline of informatics in fashion research. It also proposed ways to minimize the errors caused by subjective interpretation of experts to derive novel and generalized insights from quantitative data.

Additionally, while our findings used similar methods as previous studies (An & Park, 2020; Zhao & Min, 2019), it added new insights to fashion informatics research by including techniques such as topic modeling and sentiment analysis. From the perspective of fashion research, we proposed a new methodology for analyzing fashion collections by incorporating a qualitative analysis to traditional topic modeling techniques. This study also proposed a new tool to analyze consumers’ evaluation of the fashion brands’ marketing activities, which in turn is based on consumers’ brand sentiments. Traditional approaches for the evaluation of fashion brands using social media big data has been limited to survey methods (Heo & Lee, 2019). However, this study’s approach goes beyond surveys and financial statement analysis to provide new academic and managerial implications for evaluating the marketing performance of fashion companies. Therefore, the methods utilized in this study complement that of the previous studies on fashion design and consumer responses in that they successfully identify various design themes and brand evaluations that had not been previously explained.

The impact of SNS and fashion influencers can be explained by the trickle-down theory. Simmel (1904) theorized that people in the lower socioeconomic classes emulate the clothing and symbology of higher socioeconomic classes to achieve mobility. McCracken (1988) modified Simmel's point and claimed that rather than rich individuals, powerful and influential people are more likely to be emulated. The original trickle-down theory (Simmel, 1904; Veblen, 1899) accounts for the importance of pricing and upper classes’ attention to large fashion houses; however, McCracken’s version helps to elucidate the influence of these smaller but more powerful designers on consumer tastes (Walmsely, 2011). However, with the development of social media and the emergence of micro consumer segments who follow the influencers’ curated information, merely analyzing visual data is insufficient for explaining street fashion. Therefore, analyzing the communication generated by influencers during the fashion week can help fashion brands to predict the fashion trends with the biggest impact. In this context, this study’s empirical analysis provides meaningful insights on real street fashion.

### Practical contributions

Acknowledging that the fashion week plays an important role as a means of communication between brands and customers, analyzing consumer-driven data provides considerable value for fashion marketing and retailing. In this study, we used SNS data to determine popular brands and fashion items and gained more insights on the themes embodied by brands and collections. This method has several practical implications. For example, using our informatics methods, fashion brands and designers can use consumer-driven data to outline their strengths and weaknesses and make improvements for the next fashion week, thereby providing useful information for marketing or product planning in the fashion industry, where fashion trends and inspirations are crucial.

Moreover, each brand’s sentimental evaluation during the fashion week can be numerically cross-checked through consumers’ tweets. Twitter is one of the fastest platforms that relays consumers’ reactions, making it easy to perform numerical calculations of a brand’s sentimental evaluation, especially in comparison with other brands. We classified the consumers' Twitter reactions (through retweets, likes, and quote tweets) into three elaborate sentiment reactions (positive, neutral, and negative) where the sentimental evaluation of brand was measured at the rate of positive reviews. We also considered consumers’ tweets to measure each brand’s sentimental evaluation, making it much more reflective of the consumers’ opinions than the brand’s performance evaluations while avoiding possible biases from individual experts. This is information could help brands adapt and improve their collections for the next fashion week.

### Limitations and suggestions for future research

Although the present study offers meaningful additions to the existing literature, it has some limitations that can be addressed through future studies. First, this study’s analytical methods were conducted in English. Therefore, applying the methods used in this study to tweets written in other languages could lead to a lower classification accuracy. Since a fashion week is both a global and a regional event, it would be useful to analyze the trends through the host city’s local language. Second, this study selected the 2019 F/W Fashion Weeks as the target of analysis, which is already outdated in the marketplace. However, the essence of this study is to propose ways to analyze fashion trends through various informative approaches. Since this study analyzed the fashion weeks held in one particular year, additional stop words need to be included in the analysis of fashion weeks in other years or during the Resort/Cruise and Pre-Fall seasons.

This study’s consumer-driven data analysis approach can be repeatedly applied to data from other years (past or future), and it can confirm any long-term changes in other fashion trends. For example, this method can be used for a comparative analysis of fashion trends before and after epidemics such as SARS, MERS, and the Covid-19 pandemic. Furthermore, it can also confirm when the trends analyzed through text mining appear in real street fashion, enriching current studies on fashion trends.

Expanding upon this research, future studies could focus on developing a model that predicts the keywords or popular brands that might be frequently referenced in the fashion weeks. For example, our findings revealed certain noticeable themes such as “sustainability,” “ethical fashion,” and “street style.” Furthermore, while this study focused on the trend analysis, subsequent research can confirm whether certain topics in fashion week are associated with major stream brands’ products and whether it is possible to predict the gatekeeper effect of the fashion weeks. In terms of analytical methods, subsequent studies can investigate how Twitter data can be linked to the trends and patterns in street fashion through the simultaneous use of text mining and regression analysis. Finally, future studies could incorporate a multi-category sentiment analysis to analyze more diverse consumer responses, such as anger, happiness, sadness, and interested/not interested.

## Conclusion

This study analyzed and confirmed the city-wise characteristics of the Big 4 Fashion Weeks, the consumer’s perceptions of the designers’ clothes, and the brand’s evaluation. It also contributed to the diversification of fashion research by applying various informatics techniques used in engineering, business, and sociology to analyze fashion collections, and identified ways of applying this methodology to achieve replicable results in the field of fashion Our methodological approach also contributes toward solving the problem of objectivity that was lacking in traditional qualitative analyzes of fashion collections. Consequently, our research’s findings and methods can enable scholars to raise several interesting research questions that can deepen our understanding of the fashion phenomenon in human society. Although the current study itself may be limited in its immediate theoretical implications, its main contribution lies in its ability to stimulate more research that can advance the existing theories in future.

## Data Availability

Please contact author for data requests.
